# Microstructure and mechanical properties of Al/Cu-SS hybrid composite via ball milling and friction stir processing

**DOI:** 10.1016/j.isci.2025.114008

**Published:** 2025-11-11

**Authors:** Zikun Wang, Xianyong Zhu, Chen Wang, Xiong Xiao, Ke Zhang, Cheng Jiang, Jiaan Liu

**Affiliations:** 1 School of Mechanical and Aerospace Engineering, Jilin University, Changchun 130022, China; 2 Chongqing Research Institute, Jilin University, Chongqing 401123, P.R. China; 3 College of Materials Science and Engineering, Jilin University, Changchun 130022, China

**Keywords:** Applied sciences, Materials application

## Abstract

To balance aluminum matrix composites’ (AMCs) strength and ductility, this study used hybrid micron-sized Cu/316L stainless steel (SS) particles, fabricated via ball milling (BM) and multi-pass friction stir processing (FSP). Cu mainly enhances strength through load transfer, while the addition of SS further promotes the pinning effect and dynamic recrystallization (DRX). The *in situ*-formed Al_2_Cu further strengthens fine grain strengthening, dispersion strengthening, and load transfer efficiency. Al-5Cu AMCs showed 185.2 MPa ultimate tensile strength (UTS, +104.4% vs. matrix) but 13.8% elongation (−9.2%). Al-10SS AMCs had milder UTS (143.3 MPa, +58.2%) but better ductility (19.3%, +27.0%). The optimal Al-5Cu-10SS AMCs exhibited 183.6 MPa UTS (+102.6%), 58.96 vickers hardness (HV, +96.5%), and 20.3% elongation (+33.6%), with fracture dimples correlating to its performance. This hybrid reinforcement synergizes particle mechanisms, offering an effective route for high-performance AMCs.

## Highlights

• AMCs are fabricated by BM and multi-pass FSP
• Cu-SS hybrid particle reinforces AMCs
• A dual improvement in the strength and ductility of AMCs has been achieved

## Graphical abstract

**Figure:**
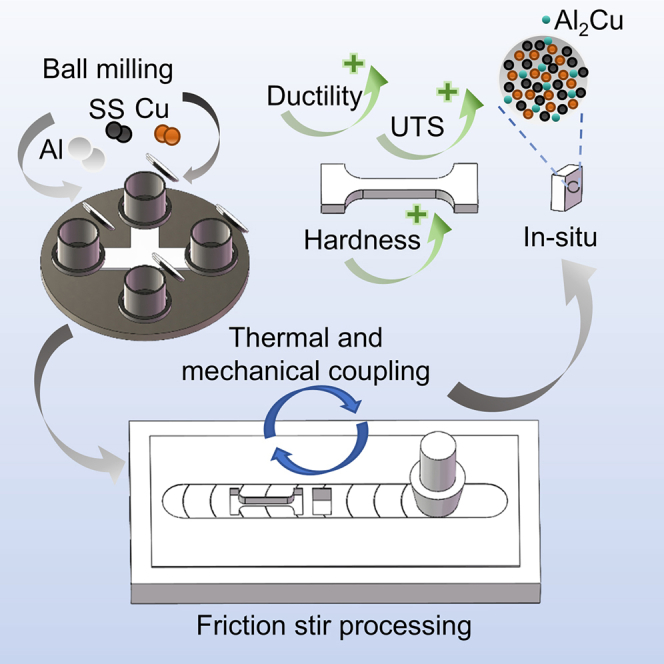

## Introduction

Aluminum alloy has the advantages of low density, good processability, and easy availability of raw materials, which has potential in lightweight, but its strength is facing challenges.^1^ Aluminum matrix composites (AMCs) have become one of the main methods to improve their strength. It is a composite with aluminum or aluminum alloy as the matrix, and its properties are improved by adding other reinforcements (such as particles, fibers, etc.). The traditional reinforcement particles usually use ceramic particles (such as SiC/Al_2_O_3_), carbon fibers, etc., to enhance their related properties.^2^ These particles have high hardness, but due to the poor wettability and interface bonding with the aluminum matrix, these AMCs are usually accompanied by a reduction in plasticity while improving their strength.^3^^,^^4^^,^^5^ How to balance strength and ductility has become an important challenge in the development of AMCs.

Metallic particles have garnered significant interest as superior reinforcement candidates in AMCs owing to their exceptional interfacial compatibility with the aluminum matrix. Unlike conventional ceramic reinforcements, metallic particles exhibit enhanced wettability and form metallurgical bonding at the matrix-reinforcement interface, thereby mitigating interfacial debonding issues commonly observed in ceramic-reinforced systems. Shahi et al.^6^ fabricated an Al/Al_3_Ni composite surface layer on a commercially pure Al substrate via six-pass friction stir processing (FSP) using pre-embedded Ni powder. The average hardness of the composite is more than 60% higher than that of the base Al matrix. Balakrishnan et al.^7^ fabricated AMCs by integrating pure iron powder into a molten aluminum matrix, followed by FSP. The thermomechanical action of FSP induced severe plastic deformation (SPD), the average grain size decreases, and the dislocation density increases, which effectively overcomes the conventional strength-ductility trade-off in particle-reinforced composites. Guo et al.^8^ fabricated pure AMCs reinforced with Ti-Al_3_Ti core-shell structured particles via powder metallurgy. The interface formed *in situ* of the composite is clean and tight, and the propagation of the nucleated cracks in the shell during deformation can be effectively inhibited by the soft Al matrix and Ti core. These metal-reinforced materials have excellent mechanical properties and undergo *in situ* metallurgical reactions with the aluminum matrix, forming intermetallic phases. In contrast to alternative metallic reinforcements, Cu enables *in situ* interfacial reactions with the aluminum matrix at reduced processing temperatures. The cost-effectiveness, favorable wettability, and superior thermomechanical stability of Al-Cu intermetallic phases further establish Cu as a preferred reinforcement element for engineering high-performance AMCs with optimized interfacial integrity.^9^ However, due to the tendency of Cu and Al to react and form brittle phases Al_2_Cu at high temperatures, it leads to the increase of strength and the decrease of ductility.^10^ Stainless steel (SS)-reinforced AMCs are characterized by exceptional mechanical properties combined with retained ductility and fracture toughness, while demonstrating superior interfacial adhesion to the AMCs due to their inherent metallurgical compatibility. SS reinforcement particles can effectively improve the toughness of composites.^11^^,^^12^^,^^13^

However, the performance enhancement of a single reinforcing particle is limited and often fails to meet multiple property requirements simultaneously. Consequently, researchers have turned to introducing different types and sizes of reinforcing particles into the matrix to overcome the constraints of a single reinforcement phase and achieve further optimization of material properties. Msebawi et al.^14^ investigated the fabrication of AMCs using a CuO-SiO_2_ mixture, employing ball milling to mitigate particle agglomeration. The study demonstrated that solid-state recycling of AMCs significantly reduces energy consumption. Tang et al.^15^ innovatively co-introduced SiC ceramic and 304 SS particles into a 6061 Al matrix, demonstrating that ductile metals can transform fracture mechanisms (from interface debonding to particle fracture), thereby overcoming the conventional trade-off between ceramic reinforcement and ductility loss. Gencer et al.^16^ fabricated solid lubricant-reinforced aluminum matrix surface composites via FSP, achieving the co-integration of Sn, Cu, and graphite into aluminum-based surface composites. This established a synergistic “soft metal + hard phase + solid lubricant” tribological system, providing a reproducible technical solution and theoretical foundation for high-performance, lightweight journal bearing manufacturing. Hybrid reinforcement particles are engineered through the physicochemical integration of two or more distinct material systems. These tailored reinforcements synergistically enhance the mechanical performance of composites via optimized stress transfer mechanisms, while simultaneously improving interfacial adhesion through chemical bonding or mechanical interlocking. Furthermore, the design of different combinations of reinforcing particles enables multifunctional capabilities, such as thermal/electrical conductivity or corrosion resistance—fabrication alongside enhanced process compatibility with conventional techniques.^15^^,^^17^^,^^18^^,^^19^ Based on this method, this experiment combines the advantages of Cu and SS metal particles to explore whether it is possible to fabricate AMCs by fabricating Cu and SS mixed reinforcement particles, thereby achieving a balanced improvement in material strength and ductility. Building on this methodology, the present study investigates the feasibility of synthesizing AMCs through the strategic integration of Cu/SS hybrid metallic reinforcements, thereby achieving a balanced improvement in material strength and ductility.

The traditional methods to fabricate ACMs, such as powder metallurgy, selective laser melting, and infiltration, may cause high energy consumption, long processing time, and high cost. FSP originates from friction stir welding. The non-consumable tool rotates at high speed in the base metal to induce frictional heat and SPD. This process enables *in situ* microstructural refinement and/or the formation of homogenously distributed reinforcing phases within the matrix, achieving tailored property enhancements without bulk melting.^20^^,^^21^ The fabrication of AMCs by FSP technology has attracted many researchers’ attention in recent years. Lee et al.^22^ fabricated Al-Fe *in situ* nanocomposites via multi-pass FSP. The FSP-induced *in situ* intermetallic reactions between Al and Fe phases yielded fully dense composites with enhanced Young’s modulus and improved tensile strength. Mehran et al.^23^ fabricated Al 1060/(Fe-Cu) hybrid surface composites via FSP, achieving *in situ* synthesis of Al-Fe and Al-Cu binary intermetallic phases, as well as Al-Fe-Cu ternary compounds, at the reinforcement-matrix interface. Liu et al.^11^ employed FSP with an innovative dual-head tool to fabricate AA6061 matrix composites reinforced with 316 SS particles, improving the microhardness, tensile strength, and corrosion resistance of the composite materials. Research findings have demonstrated that metal matrix composites reinforced with metallic particles, such as Ni, Cu, SS, and W, can be successfully fabricated via FSP technology, exhibiting enhanced ductility and strength characteristics.^12^

However, the dispersion capability of FSP diminishes progressively with higher reinforcement particle concentrations during AMC fabrication. To address this challenge, premixing of reinforcement powders becomes an essential prerequisite for achieving homogeneous microstructures. During composite material fabrication, the uniform dispersion of reinforcing particles can be achieved through powder pre-mixing, which has been demonstrated to optimize interfacial bonding, effectively inhibit detrimental interfacial reactions, and enhance subsequent processing performance.^24^^,^^25^^,^^26^ Ball milling (BM), a widely employed powder processing methodology, facilitates particle size refinement, homogeneous mixing, and interfacial modification through mechanically driven collisions, frictional interactions, and shearing actions between milling media and powder particles under controlled centrifugal forces. The implementation of high-speed ball milling (HSBM) has been demonstrated to significantly mitigate powder agglomeration phenomena while effectively preserving particle dispersion homogeneity. Furthermore, mechanical alloying effects generated during the milling process have been shown to enhance interfacial bonding characteristics through intensified particle-matrix interactions.^27^^,^^28^^,^^29^

The potential for synergistic enhancement of both strength and ductility has been demonstrated in hybrid metal particle-reinforced AMCs through previous studies. This study aims to construct a “Cu/SS” bimetallic hybrid reinforcement system in an A1060 aluminum matrix through a coupled “HSBM-multi-pass FSP” process, thereby providing both theoretical and experimental foundations for the design of high-strength and high-toughness AMCs. By integrating the unique advantages of Cu and SS in terms of distinct reinforcement characteristics, this hybrid reinforcement strategy is designed to overcome the limitations of conventional single-particle systems, ultimately achieving an excellent balance between strength and ductility in AMCs.

Based on the aforementioned methodology, AMCs were fabricated through an integrated approach combining HSBM and multi-pass FSP. The experimental matrix material was selected as A1060 aluminum alloy, characterized by its ultralow concentration of non-aluminum constituents. This compositional purity can significantly minimize interference from incidental elemental interactions during composite fabrication processes, thereby ensuring precise control over reinforcement-matrix interfacial phenomena. In the present investigation, micrometer-scale SS and Cu particulate reinforcements were subjected to HSBM pretreatment, followed by comprehensive microstructural characterization using optical microscopy and scanning electron microscopy (SEM). Phase formation mechanisms were systematically identified through energy-dispersive spectroscopy (EDS) and X-ray diffraction (XRD) analyses, and differential scanning calorimetry (DSC) was used for supplementary analysis. The mechanical properties of the composites were systematically evaluated, with subsequent analysis focusing on the influence of *in situ*-formed phases on material performance.

## Results and discussions

### Morphology and phase analysis of ball-milled powders

**Figure 1:**
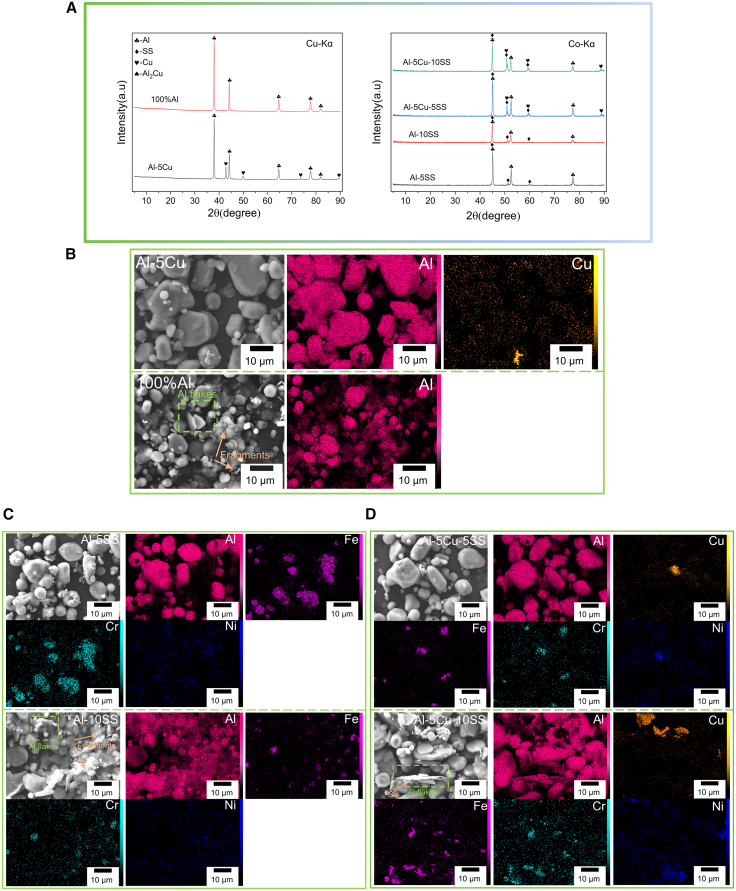
Microscopic analysis of different combinations of powders after BM (A) XRD analysis of different powder combinations under Cu-Kα target and Co-Kα target. (B–D) SEM image and EDS mapping analysis of milled powder after BM. The powders exhibit flattened and fragmented characteristics after BM. Scale bars, 10 μm.

Figure 1A presents the XRD patterns of HSBM powders with varying compositions. Specifically, pure Al and Cu powders were analyzed using a Cu-Kα radiation source (λ = 1.5406 Å). In contrast, cobalt-targeted XRD (Co-Kα, λ = 1.7889 Å) was employed for composite powders containing SS. The selection of X-ray target materials tailored to the elemental composition of samples effectively mitigates fluorescence interference while enabling enhanced detection of weak crystalline textures or low-concentration phases. As evidenced by the XRD patterns in Figure 1A, the diffraction peaks associated with distinct elemental constituents were well resolved at their characteristic 2θ positions, consistent with Bragg’s law^30^ predictions for the respective crystal structures. The inherent wavelength disparity between Cu-Kα and Co-Kα radiation sources induced systematic angular shifts in diffraction peaks corresponding to identical crystallographic planes. For instance, the (111) reflection of aluminum, which was detected at 38.0° with Cu-Kα radiation, was observed to shift to 45.0° when analyzed with Co-Kα irradiation. This angular displacement is governed by Bragg’s law, and the experimentally measured peak positions align with theoretical calculations reported in prior studies.^9^^,^^12^^,^^31^^,^^32^ As shown in Figure 1A (Al-5Cu-10SS), the diffraction peaks near 50.8° and 59.5° were markedly intensified following the sequential addition of SS and Cu powders. This enhancement is attributed to the overlapping diffraction angles of the (111) and (200) crystallographic planes for both SS and Cu. A distinct peak corresponding to the (220) plane of Cu was observed at 88.8°, while other diffraction peaks remain unchanged. These observations confirm that the HSBM process did not induce the formation of intermetallic compounds (IMCs) but merely facilitated the physical integration of distinct metallic particulates.^33^^,^^34^

The morphological evolution of HSBM Al, Cu, and SS powders is characterized by SEM and EDS analyses in Figures 1B–1D. The initial spherical Al powder underwent significant flattening (green dashed box in Figures 1B–1D) and fragmentation into smaller particles (orange arrows in Figures 1B and 1C) after HSBM, while Cu and SS powders were similarly flattened and fragmentated to varying degrees and dispersed around the Al matrix. The flattening and refinement of metal particles during HSBM are attributed to cold welding-fragmentation cycles induced by intense shear forces and compressive stresses from ball-media impacts.^35^ On the one hand, shear forces generated by the relative sliding at ball-powder interfaces disrupt reinforcement agglomerates, enhancing the uniform dispersion of composite powders.^36^ On the other hand, the compressive force applied by collisions between grinding balls and powder induces flattening of spherical Al particles, forming lamellar structures via cold welding. Subsequent fragmentation through repeated impacts ultimately refines the particle size. This cycle mechanism promotes the bonding between different particles through repeated plastic deformation and interfacial particle diffusion. The enhanced interfacial bonding in HSBM-processed mixed powder may be attributed to the formation of Al flakes (green dashed box in Figure 1D). Ultrafine Cu and SS particles are anchored onto the lamellar Al flakes through adhesion, resulting in interfaces with enhanced bonding sites for reinforcement particles. Furthermore, the particle adhesion mechanism optimizes the homogeneity of the Al-reinforcement mixture, effectively suppressing the loss of reinforcement particles during FSP.^33^

In conclusion, the homogeneous distribution of reinforcement particles and partial grain refinement can be optimized by the HSBM process. Prior research has demonstrated that the flake-like morphology of Al powder not only facilitates the adhesion and encapsulation of reinforcement particles but also promotes the formation of IMCs during the FSP stage.^34^^,^^35^^,^^37^

### Morphology and phase analysis of AMCs

**Figure 2:**
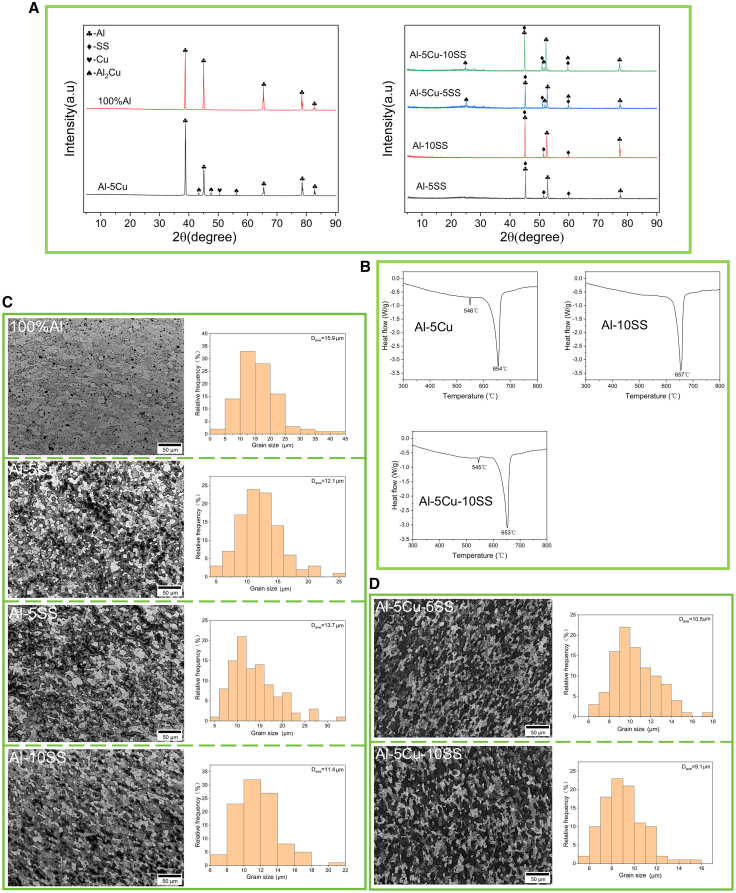
Phase and grain size analysis of the samples after FSP (A) XRD analysis of different AMCs under Cu-Kα target and Co-Kα target. It was found that the Al_2_Cu phase was generated. (B) DSC thermograms of AMC samples with different compositions and contents. An obvious endothermic peak appeared at 546°C for the Al-5Cu AMC sample, which is related to the dissolution of the Al_2_Cu phase. (C and D) The grain size distribution of AMC samples showed that the addition of reinforcing particles significantly reduced the grain size. Scale bars, 50 μm. Data are represented as mean.

The XRD patterns of the FSPed AMCs were acquired using both Cu-Kα and Co-Kα radiation sources, as illustrated in Figure 2A. The characteristic peaks of all elements detected by XRD were consistent with the previous research results.^9^ As shown in Figure 2A, the XRD patterns of the Cu-containing composite after multi-pass FSP exhibit distinct diffraction peaks corresponding to the Al_2_Cu phase (Cu-Kα: 42.6°, 47.8°, and 56.6°; Co-Kα: 24.9°, 51.8°, and 59.8°), which are absent in the HSBM powder. Concurrently, the intensity and quantity of metallic Cu peaks are significantly reduced compared to the unprocessed HSBM sample. These observations confirm that the Al matrix and Cu particles undergo an *in situ* reaction during FSP, resulting in the formation of Al_2_Cu IMCs.^38^ XRD analysis confirmed the exclusive formation of Al_2_Cu intermetallics during FSP, with no detectable phases of other IMCs. This phenomenon is likely linked to the intrinsic alloy properties of SS, as the stir zone (SZ) temperature^12^^,^^33^^,^^39^ (400°C–500°C, ∼32% of the SS melting point) remained insufficient to activate SS-Al interfacial reactions. These observations are consistent with the study of Selvakumar et al.^12^ In the FSP system of this study, the formation behavior of Al_2_Cu phase is jointly governed by thermodynamic driving force and kinetic conditions. The Gibbs free energy change (ΔG) serves as a critical thermodynamic parameter governing the feasibility of *in situ* intermetallic compound formation during FSP.^40^ A more negative ΔG value indicates stronger driving force, thereby significantly enhancing the likelihood of spontaneous reaction toward the target intermetallic phase. Due to the influence of heat input during processing and the content of elements, Al_2_Cu is the main intermetallic compound generated.^41^^,^^42^ In the Al-rich region of the Al-Cu system, Al_2_Cu exhibits the most negative ΔG, resulting in the strong thermodynamic driving force for its formation. This thermodynamic advantage makes Al_2_Cu have a strong tendency to form spontaneously during the FSP process. Under dynamic loading conditions of FSP, the synergistic effects of thermal-mechanical coupling significantly enhance atomic diffusion activity, thereby facilitating Cu migration within the Al matrix and promoting Al_2_Cu formation. Concurrently, SPD continuously fragments pre-existing Al_2_Cu particles, generating fresh interfaces that serve as nucleation sites for secondary phases.^9^^,^^43^ Furthermore, the proliferation of dislocation networks and grain boundary networks establishes accelerated diffusion pathways, providing a fast path for the diffusion of matrix and reinforcement particles.^23^

For AMCs containing 5 wt % Cu (Figure 2B), DSC experimental results demonstrate that in the 300°C–500°C range, the heat flow baseline remains stable at −0.45 to −0.65 W/g with no significant exothermic/endothermic peaks observed, indicating that no substantial phase transition, lattice reconstruction, or chemical reaction occurs in Al-Cu alloys within this temperature range. The material exhibits excellent thermal stability, consistent with the phase diagram showing no solid-state phase transformation in Al-Cu alloys at medium-low temperatures (<500°C).^38^ A sharp peak appears near 540°C, with a peak temperature at 546°C, which corresponds to the solid-state dissolution process of the Al_2_Cu intermetallic compound.^38^^,^^44^ During this process, the Al_2_Cu phase that gradually dissolves into the Al matrix absorbs heat, thus exhibiting an endothermic peak. A broadened endothermic peak with higher intensity appears in the temperature range of 600°C–680°C (peak temperature at 657°C). This phenomenon corresponds to the large-scale melting of both the aluminum matrix and residual phases (such as Al_2_Cu) that did not participate in the “first-stage melting” process. Since these constitute the primary phases of the alloy, their melting requires significantly more heat absorption, resulting in a more pronounced second endothermic peak in the DSC curve.^38^ The DSC curve of the AMC sample containing only SS (Figure 2B) exhibits a single endothermic peak without distinct double peaks, indicating the absence of significant intermetallic compound formation during this process,^12^ which aligns with XRD analysis results.

The grain structure and size distribution of the base material and AMCs are shown in Figures 2C and 2D. Compared to the base material, all AMCs demonstrated significant grain refinement in the SZ. For AMCs without Cu or SS reinforcement particles, the average grain size was 15.9 μm, with fine equiaxed grains observed. This refinement is attributed to SPD and continuous thermal input during FSP, which facilitates DRX.^9^^,^^34^ The unique shear strain generated by FSP breaks down the original grains, followed by recrystallization under elevated temperatures, ultimately forming refined equiaxed grains.^12^

Compared to the unreinforced AMCs, the Al-5Cu and Al-5SS composites exhibited reduced average grain sizes of 12.1 and 13.7 μm, respectively. However, localized variations in grain refinement were observed, likely attributed to low reinforcement content (5 wt %) and inhomogeneous particle distribution, indicating limited grain-refining efficiency of both Cu and SS particles at this concentration. When the SS content increased to 10 wt %, the grain size further decreased to 11.4 μm. This enhanced refinement is ascribed to the elevated dislocation density induced by the plastic deformation mismatch between the reinforcement particles and the matrix. Higher reinforcement content intensifies dislocation accumulation, providing greater driving force for DRX and accelerating grain fragmentation and nucleation, thereby promoting grain refinement.^43^^,^^45^

The hybrid Cu/SS-reinforced AMCs exhibited further grain refinement, with average grain sizes of 10.5 μm (5 wt % Cu + 5 wt % SS) and 9.1 μm (5 wt % Cu + 10 wt % SS). This enhancement is attributed to the synergistic pinning effect^12^ from the dispersed Cu and SS secondary phases in the aluminum matrix, which collectively restrict grain boundary migration and suppress grain growth. Notably, the superior hardness and thermal stability of SS particles amplify their pinning efficiency during DRX, effectively retarding grain coarsening. The intensified dislocation density caused by the plastic strain mismatch between hard particles and the matrix further accelerates DRX-driven grain fragmentation, resulting in a more refined microstructure at higher SS concentrations.

Compared to Cu-reinforced AMCs, the incorporation of SS particles enhances grain refinement by providing additional nucleation sites during DRX. The superior thermal stability of SS ensures sustained Zener pinning at elevated temperatures, whereas Cu particles are prone to dynamic recovery or dissolution, leading to weakened pinning efficiency. Consequently, SS addition promotes aluminum matrix grain refinement and suppresses coarsening, attributed to its persistent interfacial pinning.

**Figure 3:**
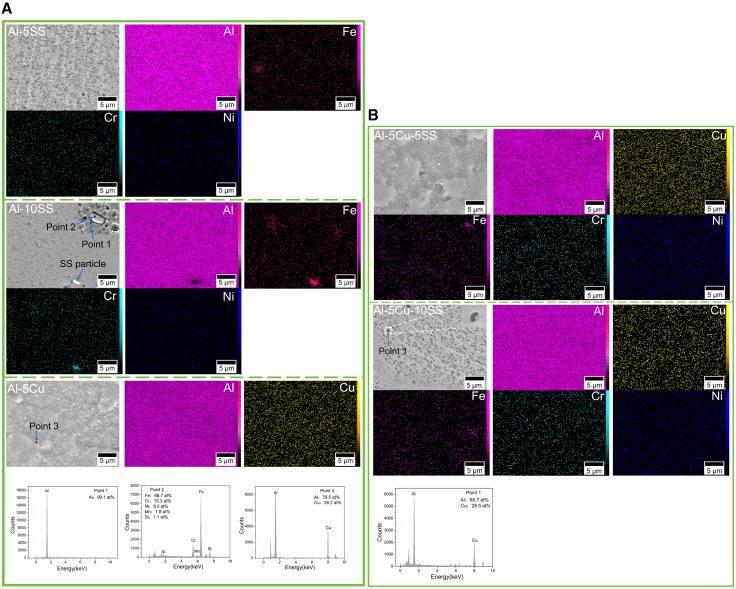
SEM image and EDS analysis of the SZ of AMCs (A and B) Building on SEM observations and EDS area mapping, additional EDS point analyses were performed at specific sites to verify the formation of Al_2_Cu. Scale bars, 5 μm.

As illustrated in Figures 3A and 3B (SEM/EDS analysis), the SZ of the AMCs subjected to multi-pass FSP exhibited a homogeneous microstructure without detectable macroscopic defects such as tunnels or cracks. EDS elemental mapping revealed that the combined effects of BM characteristics and multi-pass FSP processing enabled homogeneous dispersion of reinforcement particles.^20^^,^^21^^,^^22^^,^^36^ The localized stress concentrations were mitigated by the homogeneous dispersion of particles, while crack initiation associated with particle agglomeration was effectively suppressed. This uniform particle distribution further promoted the uniform precipitation of intermetallic phases (Al_2_Cu) during reactive processing. The formation of Al_2_Cu was confirmed through spot analysis in selected regions (Figure 3). The spot-scanning EDS analysis of selected regions in the Al-10SS sample (Figure 3A) confirmed the presence of SS particles, with no detectable secondary compound formation. This observation is fully consistent with XRD results.

In Al-5Cu composites reinforced with Cu, Al_2_Cu intermetallic phases were identified within interfacial layers surrounding dispersed particles (Figure 3A). Multi-pass FSP was observed to enhance the homogeneity of the SZ while accelerating Al-Cu interdiffusion, thereby promoting Al_2_Cu formation. In contrast, no intermetallic phases were detected in SS-reinforced AMCs, likely due to the high thermal stability of SS particles and the inability of FSP processing temperatures to exceed their reaction thresholds.^12^ The *in situ* reaction mechanism during FSP is characterized by the interaction between reinforcement particles and the matrix under thermomechanical conditions. At elevated temperatures and severe plastic strain, interfacial reactions generate layered IMCs.^9^ The intense shear forces inherent to FSP induce delamination of the IMC layers from particle surfaces, exposing fresh reactive interfaces. The fragmented IMCs are subsequently dispersed into the matrix through dynamic material flow, while the newly exposed particle surfaces re-engage in interfacial reactions due to their inherent chemical activity. This cyclical “reaction-delamination-redispersion” process progressively reduces particle size or promotes complete dissolution.

Enhanced uniformity was achieved in hybrid-reinforced AMCs compared to those containing single-particle reinforcements. The hybrid particles contributed to grain boundary pinning, thereby refining grain structures and strengthening interfacial bonding. The Cu/SS hybrid reinforcement compensated for the reduced ductility observed in Cu-reinforced AMCs. The incorporation of SS particles enabled a balanced enhancement of strength and plasticity and realized the balanced improvement of strength and plasticity.

### Mechanical properties

**Figure 4:**
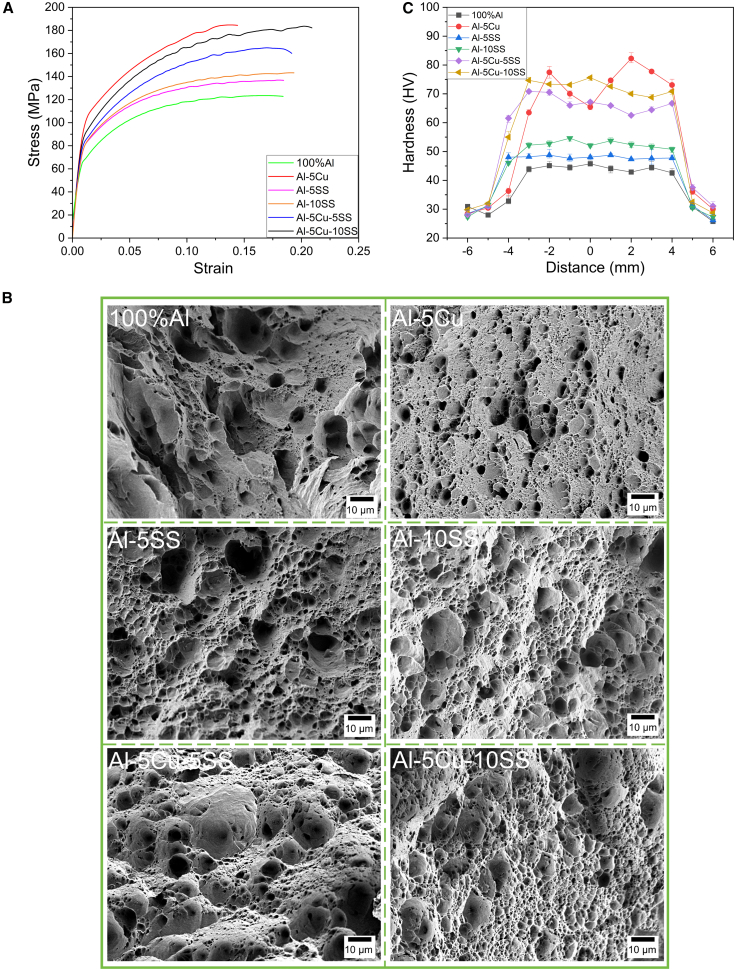
Mechanical properties and dimple characteristics of AMC samples (A) Tensile curves corresponding to different sample. The Al-5Cu-10SS sample achieved a double improvement in strength and elongation. (B) The dimples on the Al-5Cu-10SS sample were more densely distributed, with a significant reduction in size, and large and small dimples were interspersed; this was related to its superior mechanical properties. Scale bars, 10 μm. (C) The Al-5Cu-10SS sample exhibited the optimal hardness properties, which were consistent with its tensile test results. Data are represented as mean ± deviation.

The mechanical properties of AMCs were evaluated through tensile testing and Vickers hardness measurements. Representative engineering stress-strain curves for AMCs reinforced with varying particle types are presented in Figure 4A, with corresponding quantitative mechanical properties (hardness, ultimate tensile strength [UTS], and percentage elongation) summarized in Table 1. The hardness profile of the composites, measured under a 100 gf load, is shown in Figure 4C.

**Table 1:** Mechanical properties corresponding to different AMC samples

Material	Hardness/HV	UTS/MPa	El/%
Al-6061	39.2 ± 1.5	123.5 ± 1.5	17.3 ± 1
Al-5Cu	56.57 ± 3	185.2 ± 2	13.8 ± 0.4
Al-5SS	38.76 ± 2	137.2 ± 2.5	18.2 ± 0.5
Al-10SS	42.21 ± 2	143.3 ± 3	19.3 ± 0.2
Al-5Cu-5SS	51.84 ± 2.5	164.9 ± 1.5	17.1 ± 0.5
Al-5Cu-10SS	58.96 ± 3	183.6 ± 2	20.3 ± 0.4

The experimental results demonstrate that after multi-pass FSP, the 100% AMC specimens achieved UTS and elongation of 123.5 MPa and 17.3%, respectively. This performance enhancement may be attributed to FSP-induced DRX. The cyclic thermal-mechanical coupling effect of “temperature-stress-deformation” during multi-pass FSP creates ideal conditions for complete DRX occurrence, leading to grain refinement and microstructural homogenization at the microscopic level.^46^

Experimental results demonstrate that the Al-5Cu alloy with 5 wt % Cu addition exhibits an UTS of 185.2 MPa and elongation of 13.8%. While Cu particles significantly enhance mechanical strength, their incorporation compromises ductility. This is because micron-sized Cu particles can not only serve as effective load-bearing units to directly carry most of the external loads but also undergo *in situ* reactions with the aluminum matrix to generate Al_2_Cu intermetallic compounds, thereby jointly promoting a significant enhancement in the overall strength of the AMCs. However, the relatively large particle size of Cu induces localized stress concentrations during deformation, while the inherent brittleness of Al_2_Cu phases severely restricts plastic deformation capability.^9^^,^^31^ The UTS of Al-5SS and Al-10SS AMC specimens reinforced with SS particles was 137.2 and 143.3 MPa, respectively, with elongations of 18.2% and 19.3%. Although the addition of SS reinforcement particles showed less significant strengthening effects compared to copper, it achieved simultaneous enhancement of strength and ductility. This phenomenon may be attributed to the dual effects of DRX and pinning effect by SS particles during FSP, which regulated the microstructure of the matrix, refined grain size, and improved the microstructural organization.^11^^,^^12^ Consequently, both elevated strength and retained high ductility were achieved.

For hybrid-reinforced AMCs, both strength and ductility exhibited significant improvements. The Al-5Cu-5SS and Al-5Cu-10SS specimens demonstrated UTS of 164.9 and 183.6 MPa, respectively, along with elongations of 17.1% and 20.3%. Compared to the matrix, the Al-5Cu-10SS sample exhibits a 102.6% increase in UTS, a 96.5% improvement in hardness, and a 33.6% enhancement in elongation. While previous studies^11^^,^^12^^,^^15^ contributed to strength improvement, this was achieved at the expense of ductility. In contrast, the present study not only effectively enhances strength but also simultaneously improves ductility, achieving a balanced enhancement of both properties.

The simultaneous enhancement of strength and ductility may be attributed to grain refinement resulting from DRX and pinning effects. On the one hand, according to the Hall-Petch relationship,^12^^,^^46^^,^^47^ the reduction in grain size within the material can significantly enhance strength. The refined grain structure increases grain boundary density, and the greater unit volume of grain boundaries imposes more frequent obstacles to dislocation motion during slip. This complex grain boundary network effectively suppresses crack propagation, thereby enabling simultaneous improvement in both strength and ductility.^48^

On the other hand, the improvement of the performance of AMCs also stems from the effects of the Orowan mechanism and the load transfer effect. On the one hand, in AMCs, dislocation slip is typically impeded by reinforcement particles within the matrix. To continue movement, dislocations circumvent the particles, and under increasing stress, the curved dislocation lines eventually encounter each other on the leeward side of the particles, forming dislocation loops. This “particle-induced dislocation obstruction” constitutes the fundamental mechanism of the Orowan mechanism. The strengthening effect becomes more pronounced with smaller and more uniformly distributed particles, which exert greater obstruction to dislocation motion.^6^^,^^49^ The formation of Al_2_Cu precipitates acts as an effective “obstacle” during deformation, which prevents direct dislocation penetration and thereby significantly impedes dislocation motion.^31^^,^^32^ On the other hand, the load transfer strengthening mechanism facilitates the redistribution of external stresses from the matrix to high-strength reinforcements, achieving stress dispersion while mitigating localized stress concentration. This phenomenon optimizes load distribution and contributes significantly to the enhanced mechanical properties of composite materials.^49^^,^^50^ In Cu- and SS-reinforced AMCs, Cu exhibits excellent wettability with the Al, which can form *in situ* intermetallic phases Al_2_Cu through multi-pass FSP. These phases significantly enhance interfacial bonding strength between reinforcement and matrix, thereby improving load transfer efficiency.^51^ In contrast, SS particles can serve as the primary load-bearing component during deformation, while their inherent deformation capability enables partial energy absorption, mitigating localized stress concentration and thereby preventing premature fracture.^11^^,^^12^^,^^15^

The fractured surfaces of the experimental specimens following tensile testing are presented in Figure 4B. Fractographic analysis revealed that all samples exhibited characteristic ductile dimples at the fracture sites, with the fracture mode being identified as predominantly ductile failure. Notably, the geometry and distribution of these dimples (e.g., depth, diameter, and uniformity) correlate with the tensile performance metrics. The fracture surface of the 5 wt % Cu-containing specimen is characterized by a fine yet sparsely distributed dimple morphology with notable inhomogeneity, as shown in Figure 4B (Al-5Cu). This irregular dimple arrangement may induce potential stress concentration sites. This corresponds to the high strength but low ductility exhibited in the Al-5Cu sample. Although *in situ*-formed Al_2_Cu intermetallic compounds can effectively enhance the strength of AMCs, their inherent brittleness accelerates microcrack propagation under stress, leading to a reduction in elongation,^9^ which promotes cleavage-dominated fracture mechanisms. This microstructural change aligns with the strain-hardening anomalies detected in the stress-strain curves. The incorporation of SS particles induces a refined dimple structure characterized by reduced dimple size, increased depth, and homogeneous spatial distribution,^12^ as illustrated in Figure 4 (Al-5SS/Al-10SS). This morphological evolution is attributed to enhanced interfacial bonding between the reinforcing phase and aluminum matrix, facilitating effective load transfer across the matrix-reinforcement interface. The uniform dimple structure correlates with excellent tensile ductility, indicating coordinated plastic deformation during stretching and preventing premature fracture due to localized stress concentration. The elongation improvement exceeds 18% compared to the matrix material.

The dimple size of composite samples containing 5% Cu and 10% SS was significantly reduced, accompanied by an increase in dimple density. This phenomenon was attributed to the elevated density of micropore nucleation sites induced by the incorporation of higher concentration SS reinforcement particles.^13^ The high-density dimple distribution, which effectively disperses localized strain and suppresses stress concentration, enables the material to withstand higher external loads, thereby demonstrating performance consistent with its high-strength behavior. The dimple morphology transitioned from large isolated features to smaller dimples distributed around larger ones, while fracture tear lines evolved from deep and elongated to shallow and truncated. The dimple characteristics, which not only prolong the microvoid evolution period but also inhibit rapid crack propagation, enable uniform deformation and energy absorption prior to fracture, thereby achieving simultaneous enhancement of both strength and ductility.^52^^,^^53^

Not only that, during FSP, DRX was promoted by frictional heat and plastic deformation energy generated between particles and the matrix, resulting in a refined grain structure. The refinement of this microstructure effectively restricts crack propagation along the interface and complicates crack propagation paths, resulting in reduced dimple size.^54^ Furthermore, the homogeneous distribution of *in situ* phases facilitated efficient load transfer, mitigating particle agglomeration and subsequent local stress concentration.^12^^,^^13^^,^^43^^,^^45^ The aforementioned complex dimple nucleation and coalescence process effectively retards crack propagation, thereby achieving a balanced enhancement of matrix strength and ductility.

The Vickers microhardness profiles of all samples are presented in Figure 4C. Vickers microhardness measurements were performed under a 100 gf load at 12 equidistant points (1 mm spacing) symmetrically distributed across the transverse cross-section, with the weld centerline designated as the reference origin. Significant hardness enhancements were observed in both the SZ and adjacent regions of particle-reinforced AMCs relative to the base matrix, with the SZ exhibiting superior strengthening effects. The composite of 5 wt % Cu exhibited a high average hardness but displayed pronounced fluctuations within the SZ, attributed to microstructural heterogeneity, including particle clustering and localized Al_2_Cu phase segregation. In contrast, the hybrid composite with 10 wt % SS and 5 wt % Cu demonstrated superior uniformity and elevated hardness values, with a 96.5% increase compared to the substrate.

The enhanced hardness of the AMCs is primarily governed by three synergistic mechanisms: (1) grain refinement through DRX during thermomechanical processing, obeying the Hall-Petch relationship^55^; (2) composites strengthening via *in situ* Al_2_Cu phases that impede dislocation motion through Orowan mechanisms^32^; and (3) dislocation accumulation induced by coefficient of thermal expansion (CTE) mismatch between reinforcements (SS/Cu) and the Al matrix. During FSP, the CTE disparity combined with SPD promoted high-density dislocation networks, effectively hindering dislocation motion. The uniform distribution of reinforcing phases can be achieved through multi-pass FSP, which simultaneously promotes *in situ* Al_2_Cu precipitation. Enhanced hardness in the SZ is attributed to multi-mechanistic synergy, consistent with previous research reported by Azizieh et al.^32^^,^^55^

### Conclusion

The hybrid Cu/316LSS-reinforced AMCs were successfully fabricated through BM and four-pass FSP. A comparative analysis of single-particle (Cu or SS) and hybrid-particle reinforcements was conducted to evaluate their microstructure and mechanical performance. Key findings are summarized as follows.

1. BM enables both grain refinement through repeated cold welding-fracture cycles and promotes uniform dispersion of metallic particles on Al flakes. The homogeneous distribution of metallic particles facilitates the *in situ* formation of Al_2_Cu intermetallic compounds during subsequent multi-pass FSP processes.
2. All AMCs achieve grain refinement through SPD and DRX. The *in situ*-formed Al_2_Cu further enhances the pinning effect and heterogeneous nucleation, leading to additional grain refinement. The hybrid reinforcement particles demonstrate the most significant effect, with Al-5Cu-5SS and Al-5Cu-10SS composites exhibiting average grain sizes of 10.5 and 9.1 μm, respectively.
3. The incorporation of Cu particles not only enhances the strength of AMCs through load transfer but also provides effective dispersion strengthening via the Orowan mechanism from *in situ*-formed Al2Cu, resulting in an UTS of 185.2 MPa for Al-5Cu AMCs. The improvement in ductility is attributed to grain refinement from DRX and the pinning effect of SS particles. Additionally, the addition of SS particles reduces local stress concentration, thereby preventing premature localized fracture. The Al-5Cu-10SS sample exhibits superior comprehensive mechanical properties, with a 102.6% increase in UTS, a 96.5% increase in hardness, and a 33.6% improvement in elongation compared to the matrix.

Future efforts will be directed toward optimizing hybrid particle ratios and FSP parameters, with specific emphasis on post-FSP treatments to unlock higher performance thresholds and accelerate engineering-scale applications.

### Limitations of the study

Although this study discussed the influence of hybrid particle reinforcement at the micrometer scale on aluminum matrix composites, it did not involve more micro-level representations, and some conclusions were inferred based on previous literature research and existing experimental findings. Therefore, how to further explain the enhancement mechanism at the micro level will be the direction of our future research. At the same time, whether hybridization at the micrometer-nanometer dual scales will produce better results is also one of the key directions of our research.

## Resource availability

### Lead contact

Further information and requests for resources and reagents should be directed to and will be fulfilled by the lead contact, Dr. Xianyong Zhu (zhuxy@jlu.edu.cn).

### Materials availability

This study did not generate new unique reagents. All the reagents in this work were commercially available and directly used without further purification.

### Data and code availability

• The data reported in this paper will be shared by the lead contact upon request.
• This paper does not report original code.
• Any additional information required to reanalyze the data reported in this paper is available from the lead contact upon request.

## Acknowledgments

The authors acknowledge support from the 10.13039/100016694Science and Technology Development Projects of Jilin Province (no. 20250201092GX) and 10.13039/100015800Jilin Province Development and Reform Commission (2024C010-7).

## Author contributions

Z.W.: conceptualization, writing – original draft, methodology, and investigation. X.Z.: conceptualization, resources, and writing – review and editing. C.W.: methodology, formal analysis, and investigation. X.X.: validation and writing – review and editing. K.Z.: formal analysis and supervision. C.J.: formal analysis and validation. J.L.: investigation and data curation. All authors have read and agreed to the published version of the manuscript.

## Declaration of interests

The authors declare no competing interests.

## STAR★Methods

### Key resources table

**Table:** 

REAGENT or RESOURCE	SOURCE	IDENTIFIER
**Other**		

Al 1060	Yimai Aluminium Industry (Jiangsu) Group Co. Ltd	CAS# 7429-90-5
Al (powder)	Changsha Tianjiu Co. Ltd.	CAS# 7429-90-5
Cu (powder)	Shanghai Maoguo Nano Co. Ltd	CAS# 7440-50-8
316L SS (powder)	Shanghai Maoguo Nano Co. Ltd	CAS# 65997-19-5
X-ray diffraction, XRD	Rigaku	https://www.rigaku.com/products/xrd
Field-emission scanning electron microscopy, FESEM	Zeiss	https://www.zeiss.com.cn/microscopy/products/sem-fib-sem/sem.html
Energy-dispersive X-ray spectrometer, EDS	Oxford	https://nano.oxinst.com/products/sem-and-fib

### Experimental model and study participant details

This study does not include animals, human participants, plants, microbe strains, cell lines, primary cell cultures.

### Method details

In this research, commercially pure Al with dimensions of 200×60×8mm and 200×60×2 mm were used as the base plate and cover plate, respectively. The metallographic structure of the Al is shown in Figure S1, and its average grain size is 25.3μm. The relevant chemical element composition and mechanical property parameters are shown in Tables S1 and S2. This research used pure spherical Al powder (99.7% purity, supplied by Changsha Tianjiu Co. Ltd. China), SS powder, and Cu powder (99.5% purity, supplied by Shanghai Maoguo Nano Co. Ltd. China) as reinforcing particles. The pure Al, Cu, and SS powders exhibited mean particle sizes of 5.1, 2.1, and 2.5 μm, respectively, with their morphological characteristics and particle size distributions shown in Figure S2.

Based on the influence of varying metal powder contents on intermetallic compound formation and particle agglomeration, 5 wt% Cu powder and varying SS additions (0, 5, 10 wt%) were selected for fabricating AMCs, which were named as Al-5Cu-nSS(n=0,5,10). As a control, AMCs reinforced with single-type reinforcement or pure Al were fabricated under identical parameters. The composite materials were designated according to the weight percentages of Cu and SS. The composite containing exclusively pure Al powder was named 100%‌Al‌. Prior to BM, zirconia balls of different sizes and quantities (3 mm and 6 mm in diameter, with a quantity ratio of 1:3) were loaded into an agate ball mill jar. Subsequently, various powders with specific masses were weighed under a continuous argon flow to prepare formulations with different reinforcement particle contents, where the total mass ratio of balls to powders was maintained at 10:1.^35^ The addition of 1 wt% stearic acid as a process control agent effectively reduces powder surface energy, preventing cold welding and agglomeration while minimizing adhesion between grinding balls and powder particles, thereby enhancing mixing uniformity.^37^ Powders with different contents were milled by a shift-speed planetary ball mill (Model: QM-3SP4, Nanda Instrument, Nanjing, China) at a high rotation speed (200 rpm) for two hours. The optimal parameters of HSBM were based on our previous research.^56^^,^^57^ The schematic representation of the ball milling is shown in Figure S3.

Before conducting FSP, grooves with dimensions of 3 mm depth and 4 mm width were machined into the base plate using a milling machine to fill the mixed powders after BM. After filling the mixed powder into the groove, the groove was covered with a 2-mm-thick aluminum plate to prevent powder dispersion during processing.

This study employed a threaded cylindrical stir pin for FSP processing, which effectively enhanced shear deformation within the SZ and promoted metal plastic flow.^58^ The FSP tool was fabricated from H-13 steel, featuring a shoulder diameter of 17 mm and an M8×1 threaded pin (8 mm in diameter, 1 mm pitch) with a pin height of 5.8 mm. The FSP process was performed at a rotational speed of 1200 rpm, a traverse rate of 40 mm/min, with a tool tilt angle of 2.5°, the shaft shoulder pressing amount was 0.1mm, and completed in 4 passes.

In summary, the fabrication process can be outlined as follows: BM was first employed as a pretreatment step to disperse Cu and SS reinforcement particles. Subsequently, the pre-mixed powders were filled into the groove of an A1060 aluminum plate, compacted, and covered with an aluminum plate of the same material. Finally, four passes of FSP were conducted on the region filled with reinforcement particles to complete the fabrication. Figure S4 illustrates the schematic diagram of the FSP setup and the geometric configuration of the tool.

The specimens for microstructural analysis underwent sequential grinding with silicon carbide abrasive papers (400 to 3000 grit) to achieve a planar surface, followed by mirror-like finishing using a 1 μm colloidal silica suspension. After ultrasonic cleaning to remove residual particles, the samples were chemically etched with Keller’s reagent (1 ml HF, 1.5 ml HCl, 2.5 ml HNO_3_, 95 ml H_2_O) to reveal grain boundaries and phase distributions. Microstructural characterization was performed using field-emission scanning electron microscopy (FESEM; Zeiss, Jena, Germany) equipped with an energy-dispersive X-ray spectrometer (EDS; Oxford, MS, USA). Phase identification of ball-milled powders and AMCs was conducted via X-ray diffraction (XRD, Rigaku, America), and differential scanning calorimetry (DSC) was used for supplementary analysis.

The mechanical properties of both the base material and friction stir processed (FSPed) samples were evaluated through tensile testing and hardness measurements. Dog-bone-shaped tensile specimens (10 mm gage length, 4 mm gage width, and 4 mm gage thickness) and the metallographic specimens (12 mm gage length, 4 mm gage width, and 10 mm gage thickness) were extracted transversely from the SZ using electrical discharge machining (EDM). The tensile test was carried out on an electronic universal testing machine (INSTRON IN-STRON-5869, Norwood, MA, USA) at a rate of 1×10^−3^s^−13^.^22^^,^^56^ Vickers hardness was measured for 10s with a microhardness tester (HUA YIN HVS-1000A, Laizhou, China) under 100g load along the cross section of the reinforced composite. Repeat the test 10 times under the same conditions and take the average value. As mentioned above, the samples are shown in Figures S4C and 4D.

### Quantification and statistical analysis

This study uses Origin for statistical analysis or quantification.
