# Supplementary material for: Microstructure and mechanical properties of Al/Cu-SS hybrid composite via ball milling and friction stir processing
Source: iScience. 2025 Nov 11;28(12):114008. doi: 10.1016/j.isci.2025.114008 (PMC12682053; doi:10.1016/j.isci.2025.114008)
Supplement: Document S1. Figures S1–S4 and Tables S1 and S2 [file mmc1.pdf]

iScience, Volume *28*

Supplemental information

Microstructure and mechanical properties of Al/Cu-SS hybrid composite via ball milling and friction stir processing

Zikun Wang, Xianyong Zhu, Chen Wang, Xiong Xiao, Ke Zhang, Cheng Jiang, and Jiaan Liu

Supplemental Figures and Legends

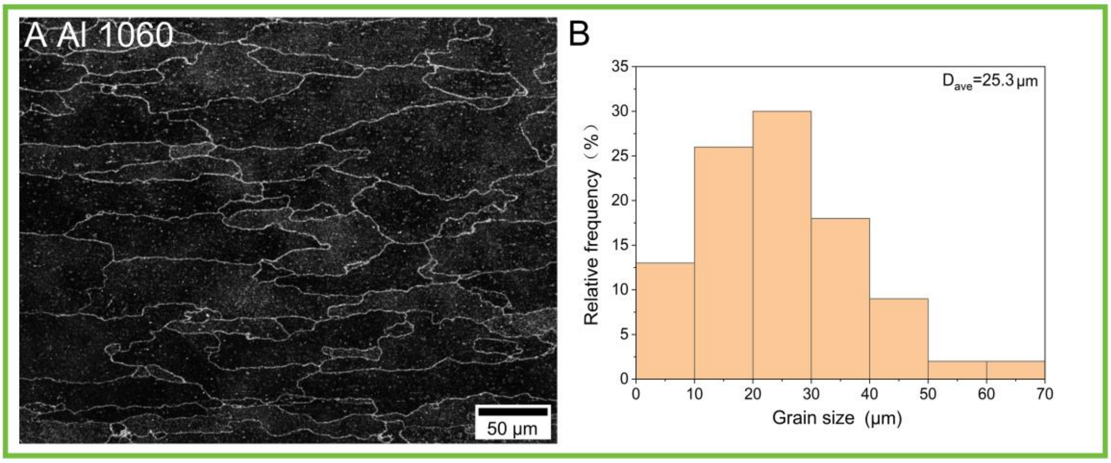

Figure S1. Grain structure and size distribution of the base material under an optical microscope, related to STAR Methods. (A) Grain morphology of the base material under an optical microscope after etching with Keller's reagent. Scale bar, 50 μm. (B) Average grain size of the base material calculated based on grain morphology statistics. Data are represented as mean.

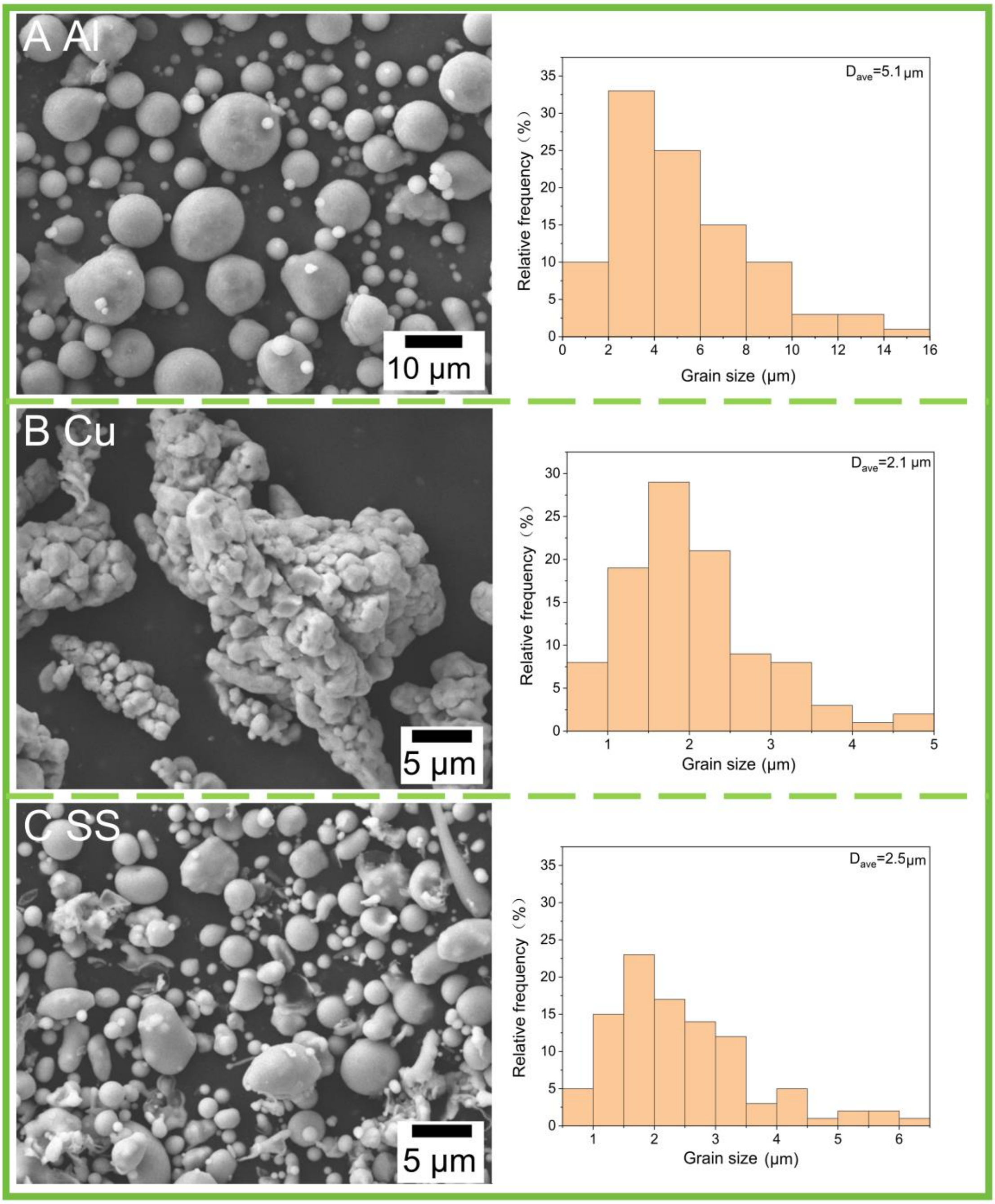

Figure S2. Microscopic morphology and size statistics of the raw material powder, related to STAR Methods. (A) SEM images and size statistics of spherical aluminum powders. Scale bar, 5 μm. (B) SEM images and size statistics of Cu powders. Scale bar, 5 μm. (C) SEM images and size statistics of SS powders. Scale bar, 5 μm. Data are represented as mean.

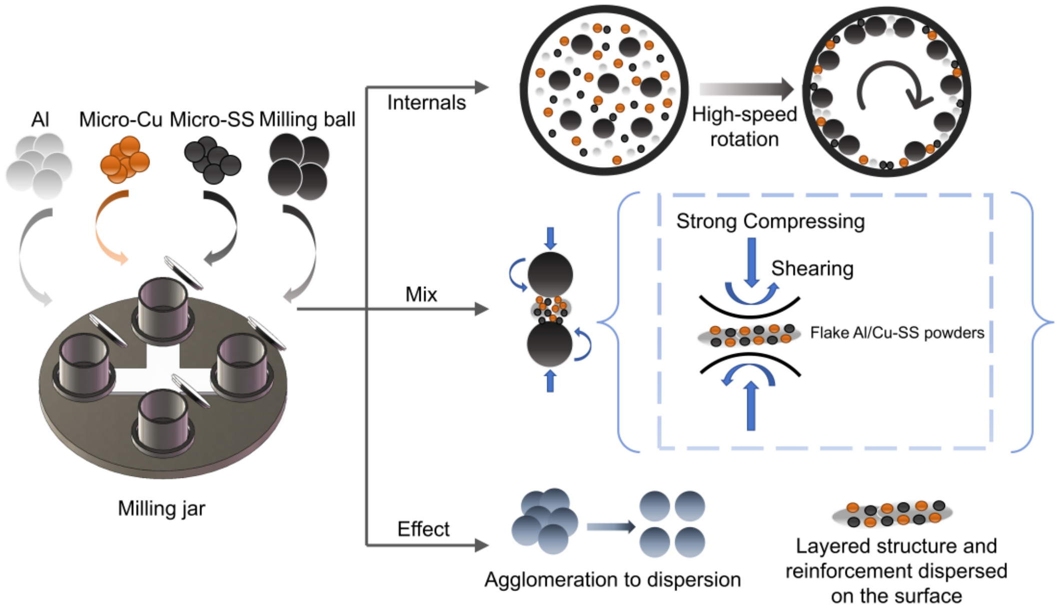

Figure S3. Schematic diagram of powder mixing via BM. BM enables efficient powder mixing, mitigates agglomeration, and facilitates the bonding between aluminum and reinforcing particles, related to STAR Methods.

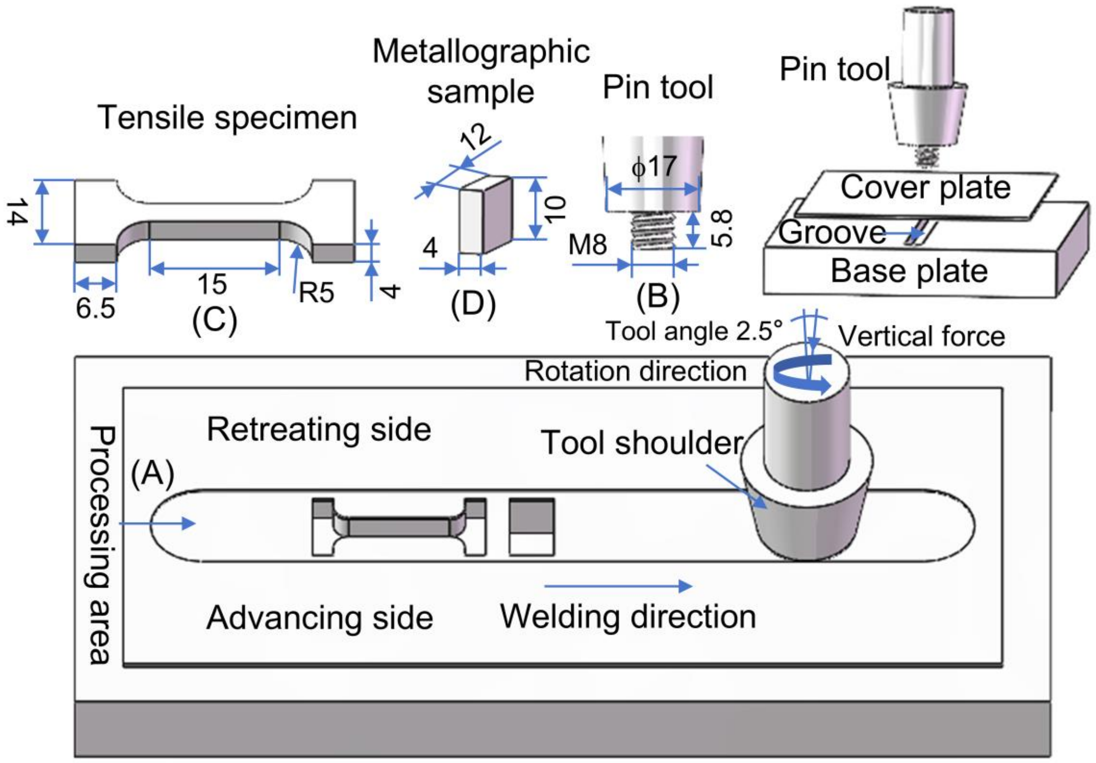

Figure S4. Schematic of FSP accomplishment, related to STAR Methods. (A) This region was filled with powders, and subsequent FSP processing was conducted. (B) A stir head with this specific size enables sufficient stirring of the processing zone, ensuring favorable forming quality of the composite material. (C) Tensile specimens extracted from the SZ. (D) Metallographic sample extracted from the SZ.

Supplemental Table

Table S1. Chemical composition of Al 1060 (wt**%**). This compositional purity can significantly minimize interference from incidental elemental interactions during composite fabrication processes, related to STAR Methods.

Materials	Mg	Cu	V	Zn	Mn	Si	Fe	Ti	Al
Al 1060	0.03	0.05	0.05	0.05	0.03	0.25	0.35	0.03	Bal.

Table S2. Mechanical properties of Al 1060. This provides a comparable basis for the fabrication of high-performance AMCs, related to STAR Methods.

Material	YS$\sigma_{0.2}$/MPa	UTS/MPa	El/%	Hardness/HV
Al 1060	54	90.6	15.2	30
